# Development and application of a highly sensitive quadruple droplet digital PCR method for simultaneous quantification of sulfonamide resistance genes

**DOI:** 10.3389/fmicb.2025.1612740

**Published:** 2025-05-21

**Authors:** Xirong Yin, Jiayuan Nie, Huifang Tian, Lihong Duan, Lixia Wu, Xiangdong Xu, Yumei Guo, Ke Wang

**Affiliations:** ^1^School of Public Health, Hebei Medical University, Shijiazhuang, China; ^2^Shijiazhuang Center for Disease Control and Prevention, Shijiazhuang, China; ^3^Hebei Key Laboratory of Intractable Pathogens, Shijiazhuang, China; ^4^The Fourth Hospital of Shijiazhuang, Shijiazhuang, China

**Keywords:** sulfonamide resistance genes, droplet digital PCR, quadruple detection, feces, sewage, surface waters, animal-derived foods

## Abstract

Sulfonamide resistance genes (*sul* genes) have a high detection rate and strong transmissibility. Therefore, there is an urgent need to develop more efficient detection methods to enhance the monitoring of *sul* genes. Current analytical methods are insufficient for the simultaneous and accurate quantification of all sulfonamides resistance genes. To overcome this limitation, a quadruple method was established by integrating droplet digital PCR (ddPCR) with the ratio-based probe-mixing strategy, achieving sensitive detection of *sul1*, *sul2*, *sul3*, and *sul4* genes in diverse matrices. Correspondingly, the primers and probes of *sul* genes were meticulously designed and rigorously validated, and the critical parameters for ddPCR such as annealing temperature, concentrations of primers and probes were systematically optimized. As a results, the quadruple ddPCR method demonstrates excellent sensitivity with limits of detection (LOD) ranging from 3.98 to 6.16 copies/reaction, and good repeatability (coefficient of variation <25%), adequately meeting the requirement for accurate *sul* genes quantification. Furthermore, this new method was applied across 115 diverse samples, including human feces, animal-derived foods, sewage and surface water, achieving positive rates of 100% for *sul1*, 99.13% for *sul2*, 93.91% for *sul3*, and 68.70% for *sul4*, with *sul* genes concentration ranging from non-detection to 2.14 × 10^9^ copies/g. In summary, the developed quadruple ddPCR method has potential to serve as an efficient and sensitive tool for monitoring *sul* genes.

## 1 Introduction

Sulfonamides, the first class of synthetic broad-spectrum antibiotics, have been widely used for treating bacterial infections and promoting growth in animal husbandry (Nunes et al., 2020). However, due to incomplete absorption in humans and animals, most sulfonamides are excreted, then enter environmental media and persist through various pathways (Spielmeyer et al., 2017). Residues of sulfonamides have been detected at concentrations as high as 107 ng/L in drinking water sources in East China (Jin et al., 2016), and up to 1,285 ng/L in urban water in the Sahara (Branchet et al., 2019). What’s more, these residues pose an unprecedented selective pressure on microbial communities, facilitating the transmission of *sul* genes in organisms and the environment (Wei et al., 2018). As a result, sulfonamides resistance has become increasingly severe, which not only strains healthcare systems but also poses serious risks to public health and threatens the long-term sustainability of ecosystems (Wu et al., 2024).

In previous studies, Yin et al. (2019) conducted a 9-year monitoring of a sewage treatment plant in Hong Kong, reporting that the positive rate of *sul* genes consistently exceeded 95%. *sul* genes are frequently employed as an indicator to assess the ARGs pollution in the environment. Enhancing the detection capacity of *sul* genes is conducive to deepening the understanding of the antibiotic-resistance issue (Haenelt et al., 2023; Felis et al., 2024). Up to now, four *sul* genes have been identified, including *sul1*, *sul2*, *sul3*, and *sul4*. Many studies showed that *sul1*, *sul2*, and *sul3* were commonly found in clinically isolated bacteria, air media, soil environments, aquatic environments, and organisms (Suzuki et al., 2019; Li et al., 2018; Pan et al., 2023). They were frequently located on mobile genetic elements (MGEs) such as plasmids, integrons, transposons and insertion sequences, which facilitated the horizontal transfer and widespread dissemination (Oliva et al., 2018; Poey et al., 2024; Pavelquesi et al., 2021). As for *sul4*, Razavi et al. (2017) first identified it in sediment samples from the Indus River, and it was confirmed to have transmissible potential. Following that, *sul4* has been detected in regions such as Southeast Asia, Europe, and China (Sharif et al., 2020; Hutinel et al., 2022; Peng et al., 2023), but comprehensive information regarding its prevalence, abundance, and host is still scant. Hence, it is highly necessary to carry out in-depth quantitative investigations on four *sul* genes.

Currently, next-generation sequencing technology (NGS), quantitative PCR (qPCR) and digital PCR (dPCR) methods have been extensively recognized as effective approaches for the detection of ARGs. NGS represents a promising and advanced methodology for monitoring ARGs, because it allows simultaneous detection of numerous ARGs along with their associated host bacteria and MGEs. In many NGS studies, the presence of *sul* genes is extremely prevalent (Lin et al., 2023; Srivastava and Verma, 2023). However, NGS technology involves highly complex operations and may miss the targeted gene (Knight et al., 2024). In contrast, qPCR and dPCR exhibit high sensitivity and specificity in the identification of ARGs. More importantly, they are capable of conducting the quantitative analysis of ARGs. Using qPCR technology, Adelowo et al. (2018) detected the *sul1* and *sul2* in the urban wetlands of Nigeria, where the concentrations spanned from 4.7 × 10^6^ to 1.2 × 10^8^ copies/g; Suzuki et al. (2013) identified *sul1*, *sul2* and *sul3* ranging from 10^−5^ to 10^−3^ copies/16S rDNA in the marine environment. Compared to qPCR, dPCR exhibits more pronounced advantages in detecting ARGs. Firstly, dPCR, featuring a lower LOD, makes it possible to detect low-abundance *sul* genes in samples (Ciesielski et al., 2021). Secondly, it can perform direct quantification without the standard curve (Su et al., 2024). Thirdly, it also shows greater tolerance to PCR inhibitors. During the experiment, the dPCR reaction system is partitioned into approximately 20,000 independent reaction units for PCR amplification, which greatly minimizes the impact of PCR inhibitors on individual units. Owing to the differences in how dPCR partitions the reaction system into independent reaction units, it can be classified into droplet digital PCR (ddPCR) and chip-based digital PCR (cdPCR) (Dong et al., 2020). Cavé et al. (2016) conducted the detection of the *sul1* in soil and organic residues with ddPCR and were able to accurately identify as low as 1.6 copies of *sul1*; Maestre-Carballa et al. (2024) also used ddPCR to quantify *sul2* concentrations in wastewater, reporting a maximum level of 1.2 × 10^5^ copies/mL. Nevertheless, based on current studies, there is a lack of methods can simultaneously quantifying all four *sul* genes.

**Figure 1:**
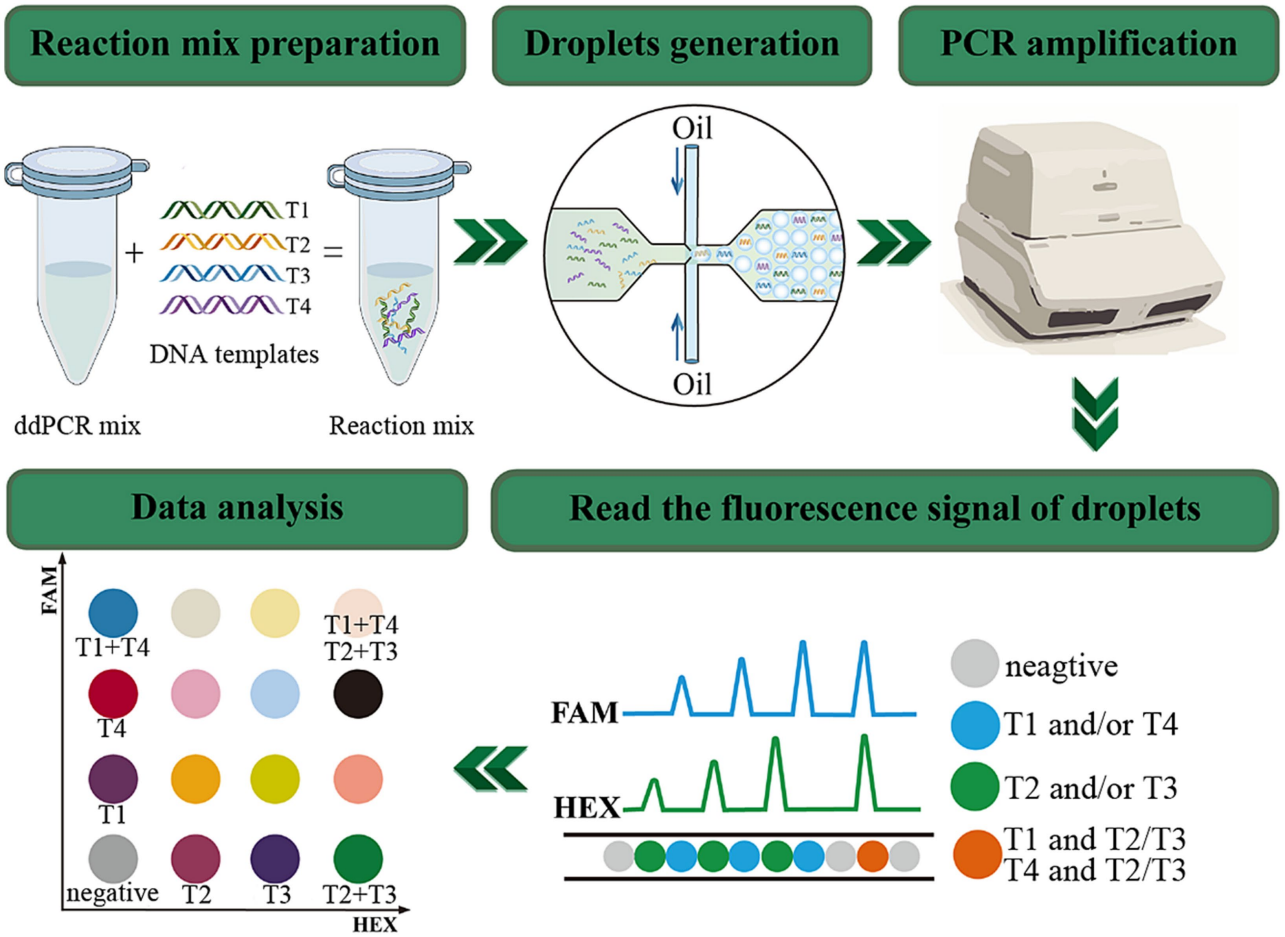
The principle and workflow of quadruple ddPCR. The FAM channel can detect signals of targets labeled with the FAM reporter group; the HEX channel can detect signals of targets labeled with the HEX reporter group. Negative droplets present as gray, the T1 and T4 labeled with FAM appear blue, the T2 and T3 labeled with HEX manifest green, and the droplets concurrently bearing FAM and HEX signals are orange.

To enhance the detection efficiency, a multiplex detection method was established by integrating the dual-channel ddPCR system with the proportion-based probe mixing strategy, so that it enables the detection of up to four target genes in a single tube (Han et al., 2022). The principle and procedure are depicted in Figure 1. In a single channel, two target genes with a significant disparity in probe concentrations coexist, and this disparity will cause a noticeable difference in fluorescence amplitude, which makes it possible to clearly distinguish between the two target gene. By selecting appropriate primers and probes combinations, Lei et al. (2020) adopted this approach developed a quadruple ddPCR assay to detect four target genes of *Vibrio parahaemolyticus* in food samples. Besides, Li et al. (2024) achieved quadruple quantification of SARS-CoV-2. These research findings demonstrate the outstanding quantification capability and potential for further development of ddPCR.

Herein, a new quadruple ddPCR method for simultaneously quantifying *sul* genes was developed with a two-channel ddPCR system (Bio-Rad, QX200^™^). Through meticulous design of primers and probes, optimization of the annealing temperature, and adjustment of the concentrations and ratios of primers and probes, this method can specifically identify and quantify the *sul1*, *sul2*, *sul3*, and *sul4* genes in samples. After that, it was applied to 115 samples, including human feces, animal-derived foods, sewage and surface water, thereby demonstrating its applicability for rapid and sensitive detection for all four *sul* genes.

## 2 Materials and methods

### 2.1 Sample collection and DNA extraction

**Figure 2:**
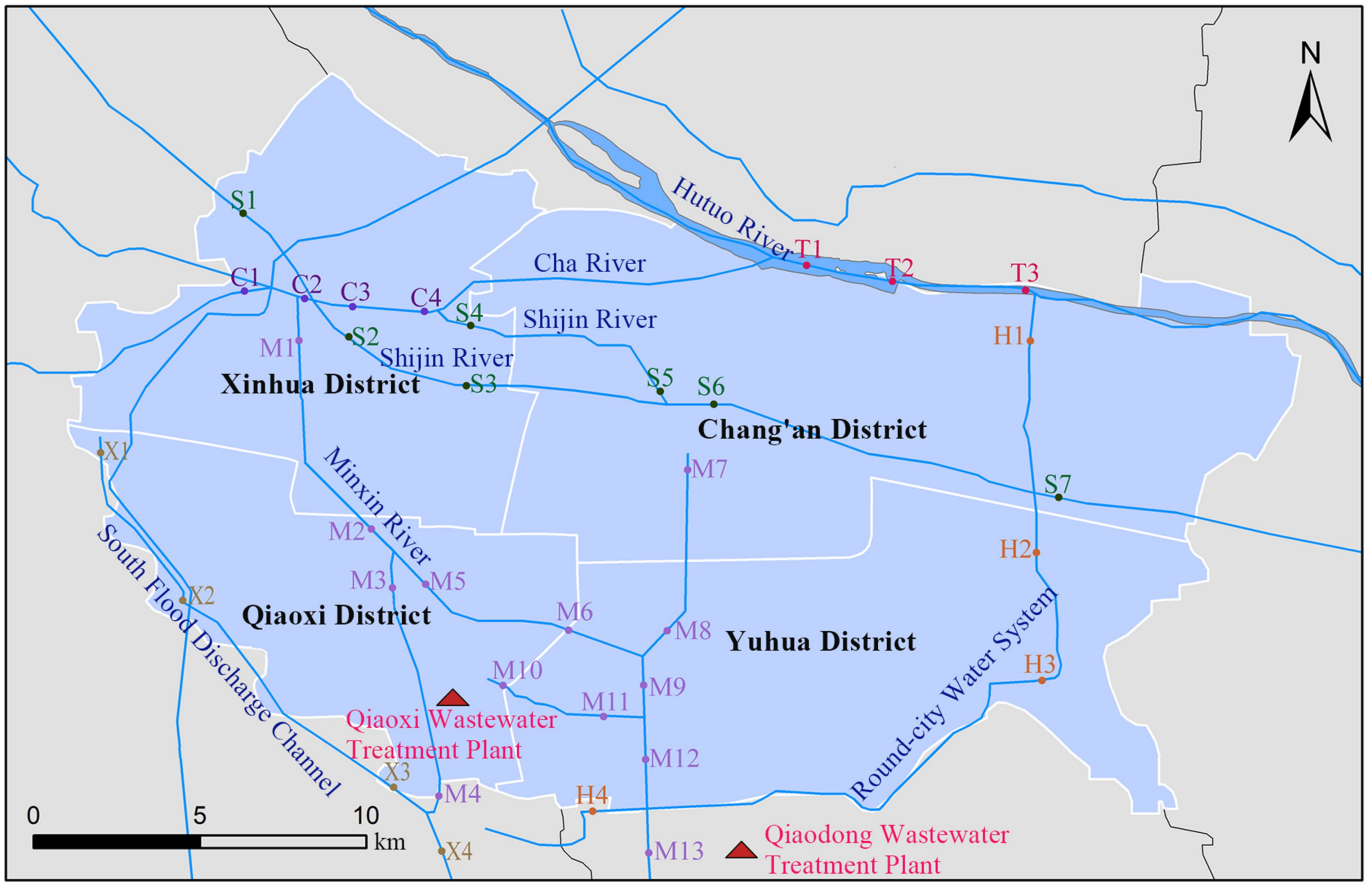
Sampling point locations of surface water and wastewater treatment plant in Shijiazhuang. C, Cha River; H, Round-city Water System; M, Minxin River; S, Shijin River; T, Hutuo River; X, South Flood Discharge Channel.

A total of 115 samples were collected in Shijiazhuang city, the capital of Hebei Province, China, including 40 human feces, 20 animal-derived foods, 20 sewage and 35 surface water. A total of 40 fecal samples from healthy humans consisted of 13 from pregnant women (gestational age 14–27 weeks), 13 from pharmaceutical factory workers (work seniority >1 year), and 14 from children (aged 0–14 years). Food samples consisted of meats (fish, shrimp, pork, and chicken) and internal organs (liver, heart), which were purchased from markets. Sewage samples were obtained from two wastewater treatment plants. As for surface water samples were collected from six rivers in Shijiazhuang city, and the sampling sites effectively covered urban areas with a high population density (the sampling points for surface water and waste water treatment plants are shown in the Figure 2). All samples were placed in disposable sterile containers and conveyed to the laboratory for pretreatment within 2 h.

The pretreatment operations for animal-derived food samples, sewage samples, and surface water samples are as follows: under aseptic conditions, accurately weigh 25 g of food samples and add them to a container containing 225 mL of physiological saline. Then homogenize the mixture thoroughly using a homogenizer (AngniInstrument, China). Transfer 10 mL of the homogenized liquid into a centrifuge tube and centrifuge it at 8,000 r/min for 10 min. After centrifugation, carefully discard the supernatant. Add 200 μL of normal saline to the above centrifuge tube. Subsequently, pipette up and down to resuspend the precipitate, which will be used as the sample for DNA extraction. As for sewage and surface water samples, 50 mL of sewage was concentrated using a vacuum filtration apparatus onto 0.45 μm filters (Millipore, United States), while 500 mL of surface water was concentrated onto 0.22 μm filters (Millipore, United States). After that, the used filters were stored at −80°C until DNA extraction was performed.

Total DNA of water samples was extracted by the DNeasy PowerWater Kit (Qiagen, Germany). PowerFecal Pro DNA Kit (Qiagen, Germany) was employed for the DNA extraction of animal-derived foods and human fecal samples, the amount of each sample is approximately 200 mg. Eventually, the concentration and quality of DNA were determined via a NanoDrop 2000 spectrophotometer (Thermo Scientific, United States).

### 2.2 Design and validation of primers and probes

The primer-probe sets of *sul1* and *sul2* were reported in previous studies (Han et al., 2021). However, the primer-probe sets for *sul3* and *sul4*, which can be amplified under the same conditions as *sul1* and *sul2* were absent. Therefore, based on a comprehensive consideration of the characteristics of the existing primers and probes, the Oligo7.6 software (Molecular Biology Insights Inc., United States) was used to design the corresponding primers and probes according to the conserved regions of the reference sequences of *sul3* and *sul4*. By leveraging nucleotide BLAST, the specificity analysis of amplicons was executed preliminarily. In addition, the primer and probe sequences and relevant information for quadruple ddPCR are listed in Table 1, and all primer and probe sets used in this experiment were synthesized by the Takara Biotech (Beijing, China).

**Table 1:** The primers and probes for quadruple ddPCR.

Genes	Gene bank number and position	Primer/probe	Sequence 5′–3′	Product size (bp)	References
*sul1*	JF969163.1; 1,054–1,893	*sul1*-F	CCGTTGGCCTTCCTGTAAAG	67	Han et al. (2021)
		*sul1*-R	TTGCCGATCGCGTGAAGT		
		*sul1*-P	FAM-CAGCGAGCCTTGCGGCGG-BHQ1		
*sul2*	AY055428.1; 20,269–21,084	*sul2*-F	CGGCTGCGCTTCGATT	60	Han et al. (2021)
		*sul2*-R	CGCGCGCAGAAAGGATT		
		*sul2*-P	HEX-CGGTGCTTCTGTCTGTTTCGCGC-BHQ1		
*sul3*	NZ_NIYS01000141.1; 2,098–2,889	*sul3*-F	AGGCTTGGCAAAGTCAGATTG	68	This study
		*sul3*-R	TAGTAGCTGCACCAATTCGC		
		*sul3*-P	HEX-ACTTGTGTTGATGCACTCCGTT-BHQ1		
*sul4*	NG_056174.1 1–1,064	*sul4*-F	CGCGCAAATCATTTATTGGCTA	68	This study
		*sul4*-R	GGCCGCCGTTCCTTCTA		
		*sul4*-P	FAM-ACGCTCGATTTGCCGCCAGACCAG-BHQ1		

F, forward primer; R, reverse primer; P, probe; FAM, carboxyfluorescein; HEX, hexachlorofluorescein; BHQ1, black hole quencher 1.

To further verify the specificity of method, some strains were isolated from pig liver, chicken heart, fish viscera and sewage samples. Their resistance phenotype were determined through antibiotic sensitivity testing. Next, the nucleic acids of strains were extracted using the boiling method. Finally, the *sul* genes of 20 strains were detected simultaneously by means of PCR, qPCR and ddPCR (the information of PCR and qPCR is presented in Supplementary Tables 1, 2).

### 2.3 Procedure of the quadruple ddPCR

For quadruple ddPCR assays, the PCR mixture consisted of 5 μL 4 × ddPCR multiplex supermix for probes (Bio-Rad, United States), 0.7 μL each forward and reverse primer (20 μM), 0.4 μL *sul1* probe (10 μM, FAM-labled), 0.4 μL *sul2* probe (10 μM, HEX-labled), 1.0 μL *sul3* probe (10 μM, HEX-labled), 0.9 μL *sul4* probe (10 μM, FAM-labled), 2 μL DNA sample and 4.7 μL nuclease-free water. Later, the well-mixed reagent was transferred to the DG8 Cartridge (Bio-Rad, United States), and placed it into the QX200^™^ Droplet Generator (Bio-Rad, United States). A total of 20 μL of reaction mixture containing the DNA template was partitioned into around 20,000 droplets. Afterward, droplets were transferred into 96 well plate and then placed in the C100^™^ Thermal Cycler (Bio-Rad, United States) for amplification. The thermal cycling conditions were set to run for 10 min at 95°C for predenaturing, 40 cycles of 94°C for 30 s, 56°C for 30 s and 98°C for 10 min to deactivate the enzyme, whole steps with a ramp rate of 1.5°C/s. Following the cycling, it is recommended that each reaction be incubated at 4°C for at least 30 min in to stabilize droplets. Next, the 96-well plate is loaded into the QX200^™^ reader (Bio-Rad, United States), the reader instrument analyzes the fluorescence signals of the droplets individually based on a two-color fluorescence channel. Ultimately, all data were processed using QuantaSoft Analysis Pro software1.0.596 (Bio-Rad, United States).

### 2.4 Limits of detection, dynamic range and repeatability

The reference sequences of *sul1*, *sul2*, *sul3*, and *sul4* were separately inserted into four pMD19-T plasmids (with a total sequence length of 2,692 bp each), and there are no other exogenous genes. All plasmids were designed and synthesized by Takara Biotech (Beijing, China). The concentrations of the four linearized plasmid solutions used in this experiment are 5 × 10^8^ copies/μL. To obtain a mixed plasmid solution with a concentration of 5 × 10^7^ copies/μL, 10 μL each of the four plasmid solutions were mixed with 60 μL of nucleic acid-free water. This solution was then serially diluted 10-fold, the concentrations ranging from 10^0^ to 10^7^ copies/μL. Subsequently, the gradient diluted plasmid solution was detected by quadruple ddPCR method (3 repetitive experiments and three technical replicates, *n* = 9), the average copy number, standard deviation, and coefficient of variation were calculated. To determine the reliable dynamic quantification range of quadruple ddPCR, the quantitative curves of *sul* genes were constructed, log_10_ (theoretical concentration of the standards) as the x-axis and log_10_ (copies/reaction by ddPCR) as the y-axis, the linear fitting coefficient (*R*^2^) was calculated by Origin 2024 (OriginLab, United States). Referring to the guidelines of the Clinical and Laboratory Standards Institute (CLSI), the LOD and 95% confidence interval (95% CI) of the ddPCR were estimated by means of the Probit approach, and the analysis was executed by SPSS 21.0.

### 2.5 Estimation of the concentration of *sul* genes in diverse samples

It must be pointed out that the concentration data acquired from ddPCR merely stand for the concentration of *sul* genes in 2 μL of the template DNA. Therefore, the following formula was developed to calculate the actual concentration of *sul* genes in the sample.


$$C=N_{\text{mean}}\times \frac{V_{1}}{V_{2}\times B\times d}\times Μ$$

*C* represents the copy number of samples (copies/mL or copies/g). *N* is the average copy number of target genes in 20 μL ddPCR system, three technical replicates are performed for each sample (copies). *V*_1_ is the final constant volume of DNA extraction (μL). *V*_2_ is the volume of template DNA in ddPCR reaction system (μL), and *B* is the volume or mass of the homogenized sample consumed during DNA extraction (mL or g), d is the dilution coefficient of the sample during DNA extraction, for instance, if the sample is diluted at a ratio of 1:10, it would be designated as 10^−1^, *M* is the dilution factor of the DNA template before its addition to ddPCR.

## 3 Results

### 3.1 Validation of primers and probes

**Figure 3:**
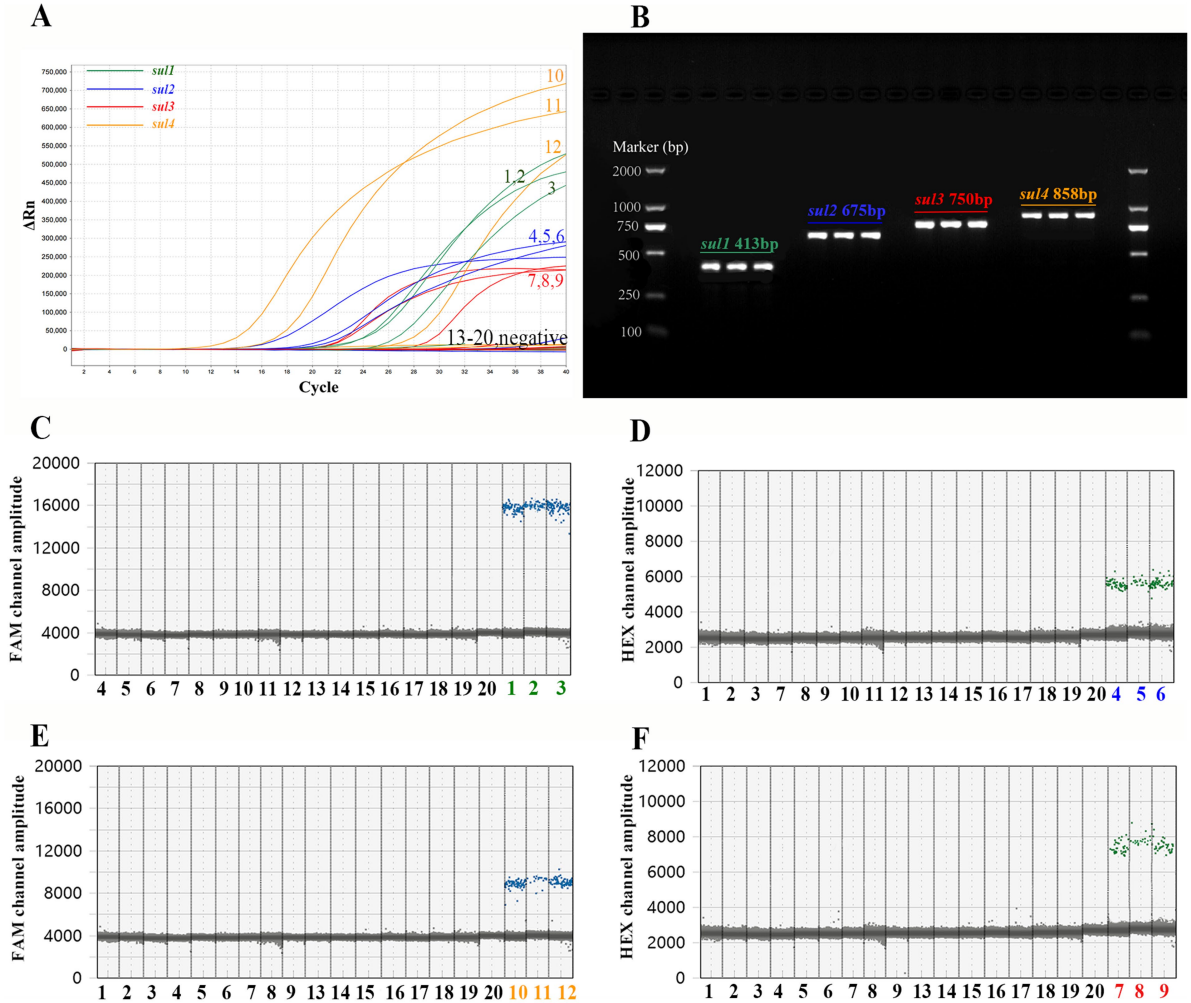
Comparison of the outcomes of PCR, qPCR, and ddPCR in the detection of actual samples. Among the 20 samples, samples numbered 1 to 3 are positive for *sul1*, samples 3 to 6 are positive for *sul2*, samples 7 to 8 are positive for *sul3*, samples 9 to 11 are positive for *sul4*, and samples 12 to 20 are negative samples. **(A)** The amplification curve of qPCR. **(B)** The agarose gel electrophoresis graph of PCR. The 2 kb DNA marker (TIANGEN, China) are on both sides of the picture, and the PCR products of the actual samples are in the middle. Text in the figure records the name of *sul* genes and the expected length of the PCR product. **(C)** Detection results of *sul1* by single-target ddPCR. **(D)** Detection results of *sul2* by single-target ddPCR. **(E)** Detection results of *sul4* by single-target ddPCR. **(F)** Detection results of *sul3* by single-target ddPCR.

As depicted in Figure 3, 20 samples were subjected to detection using PCR, qPCR, and ddPCR methods. The results obtained from these three methods showed striking consistency. More precisely, three *sul1*-positive, three *sul2*-positive, three *sul3*-positive, three *sul4*-positive, and eight negative samples were identified. The selectivity and specificity of the method employed are compellingly validated.

Subsequently, the PCR and qPCR products were sent to Beijing Tsingke Biotech Company (Beijing, China) for Sanger sequencing (ddPCR is a terminal detection technique, and its products cannot be recovered for sequencing). Finally, the sequencing results were compared with the reference sequence of *sul* genes by Snapgene software (Beijing, China). The sequence of PCR and qPCR products showed a highly matching with the reference sequence (Supplementary Figure 2), which demonstrated that all primers and probes in the experiment successfully amplified the target gene fragment.

### 3.2 Development and optimization of the quadruple ddPCR method

Generally, droplets carrying target genes can generate fluorescence signals, and such droplets are defined as positive droplets; on the contrary, droplets that do not carry target genes are called negative droplets. Obviously, the core point for ddPCR to achieve precise quantification is the ability to clearly distinguish positive droplet clusters carrying different target genes from negative droplet clusters. Hence, factors that can affect the fluorescence amplitudes of positive droplet clusters, such as annealing temperature, primer concentration, probe concentration and ratio, should be taken into consideration.

In the single-target ddPCR assay, the annealing temperature, along with the concentrations of primers and probes, on its fluorescence intensity were explored. First of all, a series of temperature gradients (ranging from 55°C to 62°C) was set, with the initial primers/probe concentrations of each target at 900 nM/250 nM (recommended by Bio-Rad). When the annealing temperature exceeded 59.5°C, the fluorescence amplitude of *sul1*, *sul2* and *sul3* decreased with the increase of temperature, while the fluorescence amplitude of *sul4* did not change (Supplementary Figures 3A–D). Preliminarily, the annealing temperature range of the ddPCR method was set from 55°C to 59.5°C. Secondly, the relationship between primer concentrations and fluorescence amplitude was investigated. When the probe concentration was fixed at 250 nM, within the primer concentration range of 500 nM to 1,300 nM, the fluorescence amplitude in the FAM channel exhibited a slight increase as the primer concentration rose. However, this change in the HEX channel was scarcely noticeable (Supplementary Figures 3E,F). This could be due to the fact that the primer concentration was excessive relative to the probe concentration. Furthermore, when the primer concentration was 900 nM, whether for FAM-labeled or HEX-labeled probes, fluorescence amplitude augmented with probe concentration in the range of 125 nM to 675 nM (Supplementary Figures 3G,H). Evidently, the probe concentration significantly affects the fluorescence amplitude of droplet clusters, and selecting suitable probe concentrations is crucial for the development of multiplex ddPCR.

**Figure 4:**
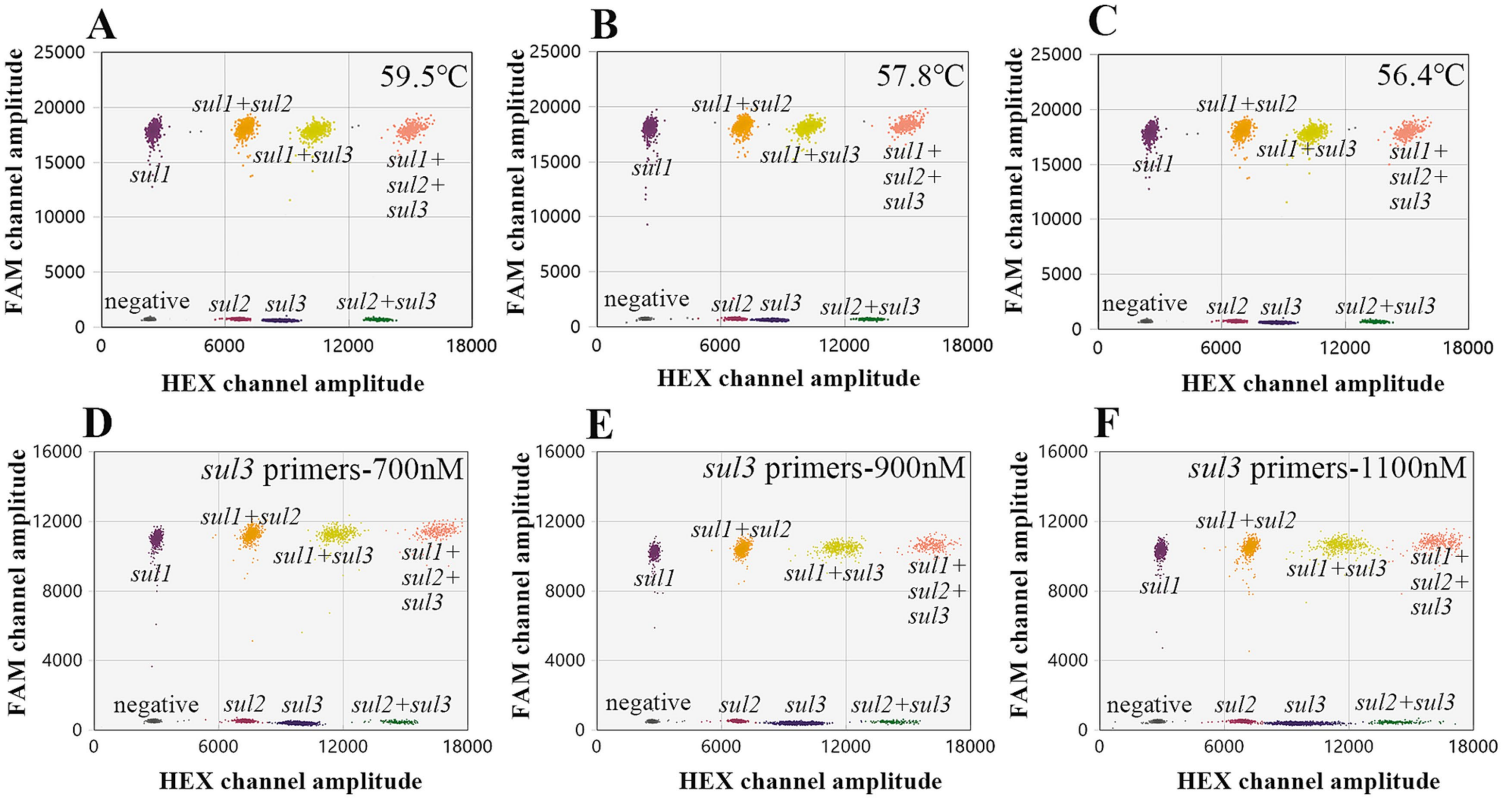
Optimize the triple ddPCR system. **(A–C)** Represent the outcomes of optimizing the annealing temperature. **(D–F)** Show that varying *sul3* primer concentrations affects droplet cluster separation efficiency.

On this basis, *sul2* and *sul3* (HEX-labeled) were set in HEX channel with the primers/probe concentrations of 900 nM/250 nM and 900 nM/500 nM, *sul1* (FAM-labeled) was set in FAM channel with the primers/probe concentrations of 900 nM/250 nM. Similarly, a temperature gradient series ranging from 55°C to 59.5°C was established. The results indicated that the droplet cluster separation efficacy was optimal within the temperature range of 55°C to 57.8°C (Figures 4A–C). Taking the stability of the instrument into comprehensive consideration, 56°C is finally determined as the optimal annealing temperature. Subsequently, the concentration of the primers were also adjusted. Taking the *sul3* primer as an example, as the concentration of the *sul3* primers rose, the dispersion effect of droplet clusters showed a notable decline. Specifically, when the primer concentration was 700 nM, the separation effect was the best (Figures 4D–F). In conclusion, the conditions of the optimized triple ddPCR method are as follows, the annealing temperature is 56°C, the primer concentration is 700 nM, and the concentrations of probes *sul1*, *sul2*, and *sul3* are 250 nM, 250 nM, and 500 nM, respectively.

**Figure 5:**
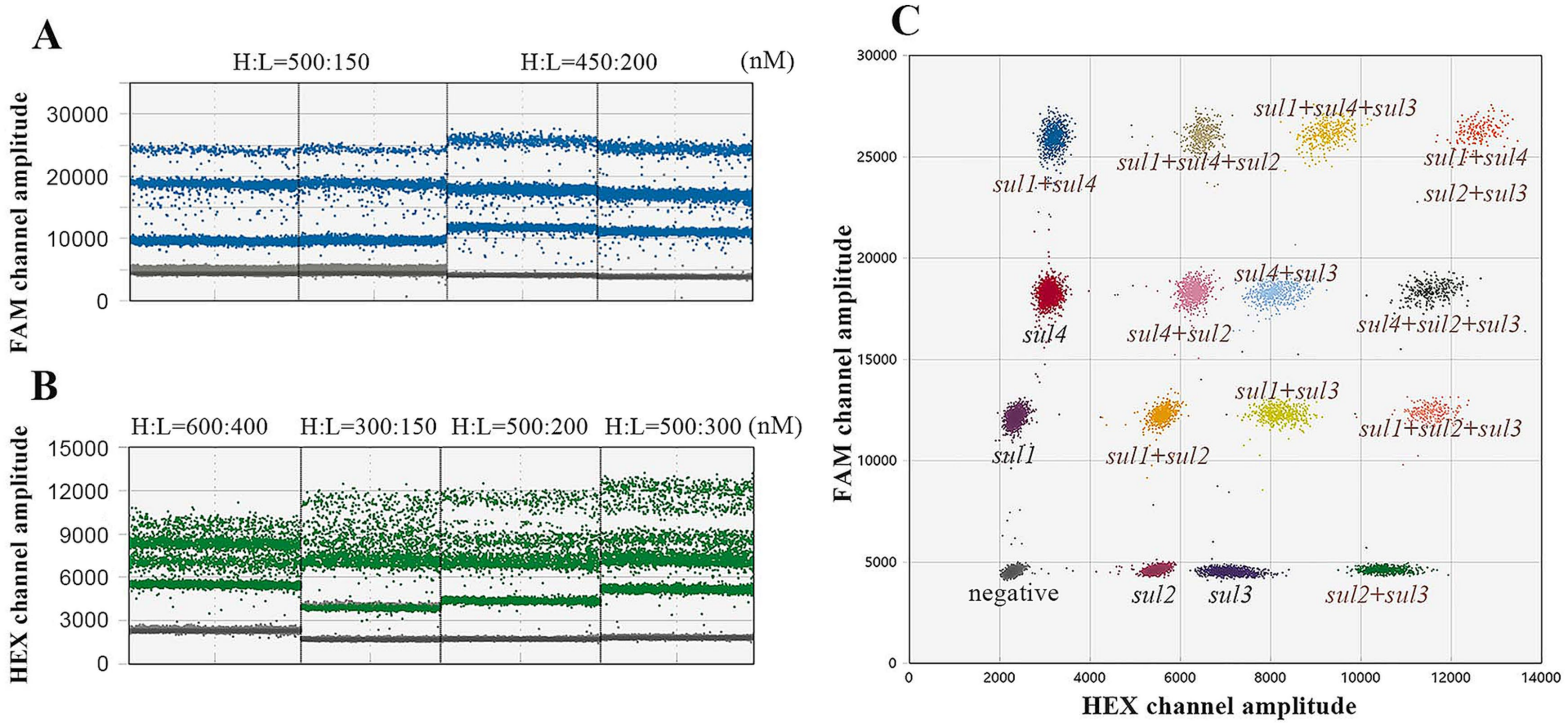
Optimize the quadruple ddPCR system. **(A)** Different probe concentration combinations of *sul1* and *sul4*; H represents the probe with high concentration and L represents the probe with low concentration. **(B)** Different probe concentration combinations of *sul2* and *sul3*, H represents the probe with high concentration, and L represents the probe with low concentration. **(C)** The 2D plot of optimized quadruple ddPCR.

In the quadruple ddPCR assay, 2^4^ droplet clusters (a total of 16 droplet clusters) are generated, including one negative droplet cluster, four droplet clusters with only a single target gene, and nine additional clusters, these additional clusters contain any two or even more target genes simultaneously. To achieve an ideal separation effect of droplet clusters, the concentrations and ratios of primers and probes were further optimized. Figures 5A,B show the optimized concentrations of FAM-labeled probes for *sul1* and *sul4* are 200 and 450 nM respectively; and the optimized concentrations of HEX-labeled probes for *sul2* and *sul3* are 200 and 500 nM, respectively. In this way, as depicted in Figure 5C all droplet clusters could be successfully dispersed.

### 3.3 Limits of detection, quantification range and repeatability of the quadruple method

**Figure 6:**
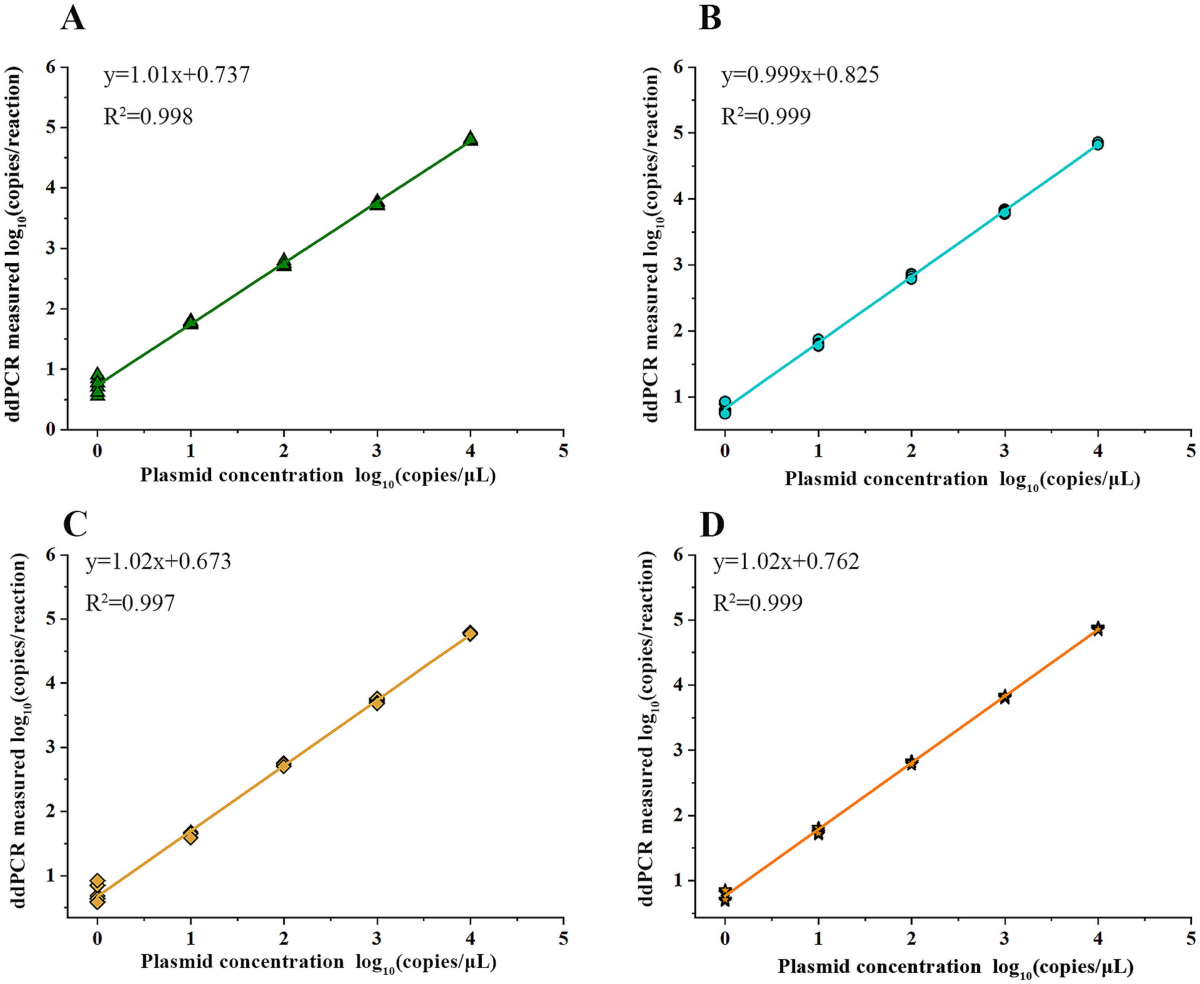
Quantification linearity of quadruple ddPCR: the x-axis represents the concentration of serially diluted linearized plasmid solutions and the y-axis represents the detection results of quadruple PCR. The linear regression equation and the correlation coefficient (*R*^2^) are shown. **(A)** *sul1* gene. **(B)** *sul2* gene. **(C)** *sul3* gene. **(D)** *sul4* gene.

The mixed plasmid solution of known concentration (5 × 10^1^ copies/μL) was serially diluted in a 5-fold manner, resulting in five concentration gradients. Then, the above-mentioned solutions were employed as DNA templates for the quadruple ddPCR, and each concentration had 12 replicates. The number of positive cases for each concentration was recorded, and the LOD and 95% confidence interval were obtained by using the Probit model (Table 2). Furthermore, to clarify the reliable quantification range of the quadruple ddPCR, it was used to measure a mixed plasmid solution at five concentration gradients (10^0^ to 10^4^ copies/μL), and the quantitative results were linearly fitted with the theoretical concentrations. As shown in Figure 6, the x-axis represents log_10_ (plasmid concentration), while the y-axis representslog_10_ (copies/reaction) acquired from the quadruple ddPCR method, each *sul* gene exhibited good linear correlation (*R*^2^ > 0.990).

**Table 2:** The LOD and 95% CI of *sul* genes.

*sul* genes	LOD (copies/reaction)	95% CI
*sul1*	3.98	(3.18, 5.16)
*sul2*	6.16	(4.31, 9.85)
*sul3*	5.77	(4.55, 9.14)
*sul4*	4.85	(3.41, 5.93)

### 3.4 Detection of *sul* genes in diverse samples

**Figure 7:**
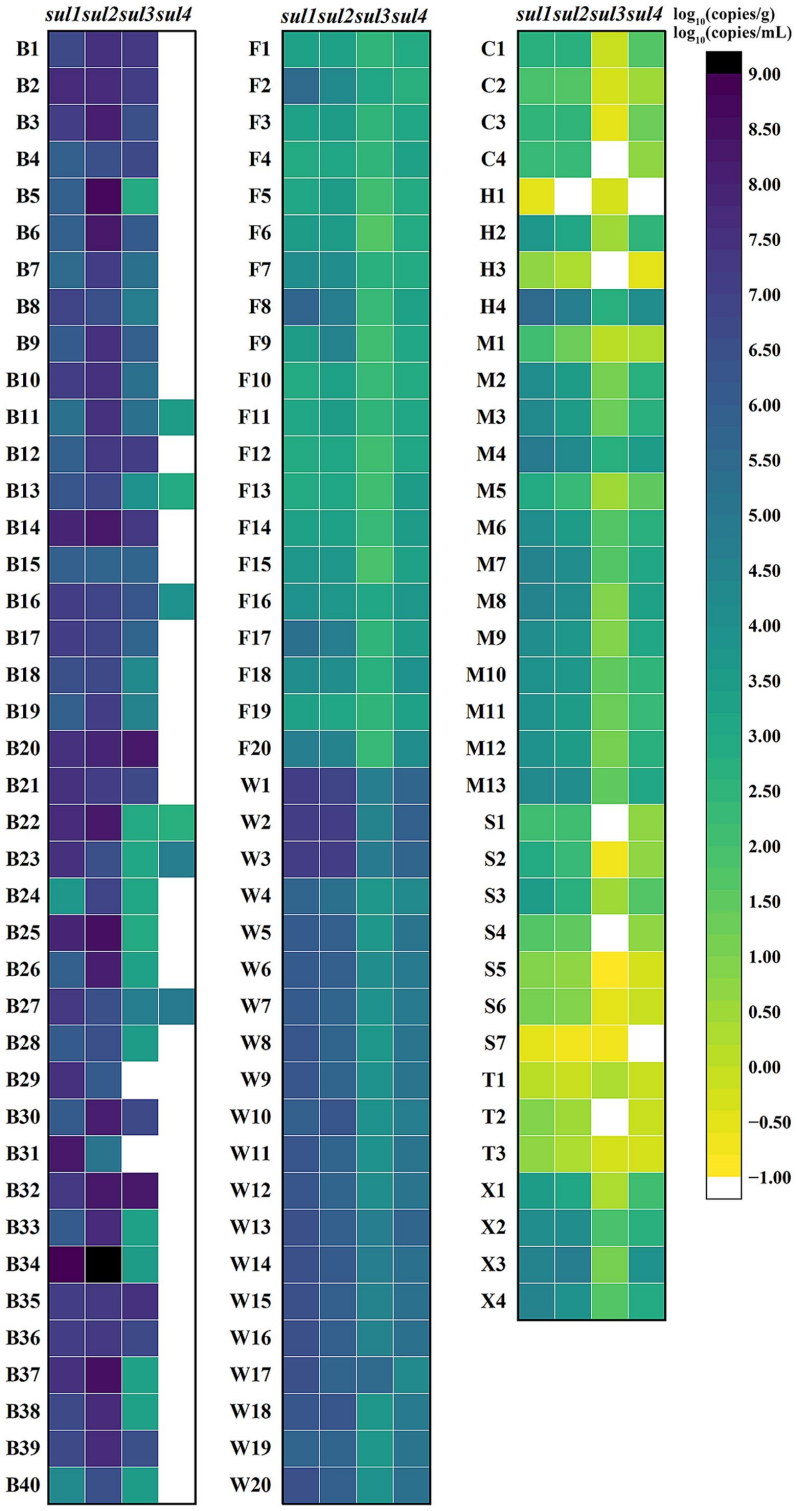
Logarithm of *sul* genes concentration in various samples. B1–B40 are human feces samples; F1–F20 are animal-derived food samples; W1–W20 are sewage samples; C1–C4 are surface water samples from Cha River; H1–H4 are surface water samples from Round-city Water System; M1–M13 are surface water samples from Minxin River; S1–S7 are surface water samples from Shijin River; T1–T4 are surface water samples from Hutuo River; X1–X4 are surface water samples from South Flood Discharge Channel. For each sample, the higher the concentration, the darker the color, and white indicates no detection.

**Figure 8:**
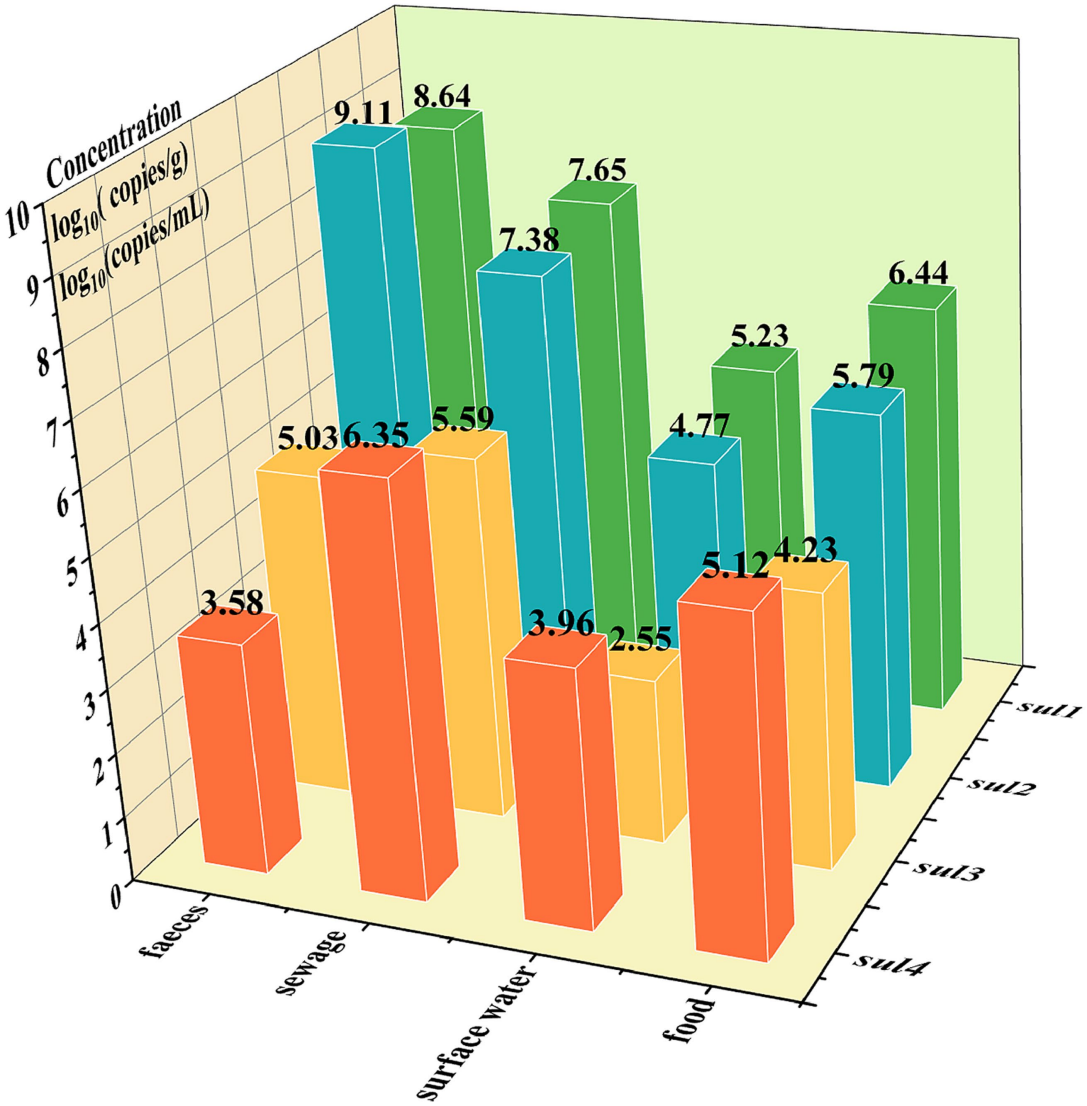
The logarithm of the average concentration of *sul* genes across different samples: the x-axis represents the sample types, the y-axis represents the *sul* gene names, and the z-axis represents the logarithmic values of average concentrations, log10 (copies/g) or log10 (copies/mL).

The quadruple ddPCR method was employed for detecting *sul* genes in 115 samples. Among the 40 human feces samples, *sul1* and *sul2* had positive rates of 100%, while the positive rate of *sul3* was 95% and that of *sul4* was 15%. For 35 animal-derived food samples and 20 sewage samples, the positive rates of four *sul* genes were all 100%. As for 35 surface water samples, the positive rates of *sul1*, *sul2*, *sul3*, and *sul4* were 100, 97.14%, 85.71, and 94.29%, respectively. To visualize the *sul* genes content in each sample, a logarithmic transformation was carried out, and color intensity was used to represent the concentration level (Figure 7). The positive frequency of s*ul4* in human fecal samples was notably low, showing a significant difference compared to other sample types. In the surface water samples, notable internal differences are manifested, which were shown by some samples not containing either *sul3* or *sul4*. To further explore the concentration differences of *sul* genes in various sample, this study calculated the average concentration of the four *sul* genes in four sample categories (Figure 8). Across various samples, the average concentration of *sul* genes was highest in human feces, followed by sewage, animal-derived foods, and surface water. The average concentrations of *sul1* and *sul2* were extremely high in human feces, reaching 1.29 × 10^9^ copies/g and 4.37 × 10^7^ copies/g, respectively. Compared with other types of samples, they were 1 to 4 orders of magnitude higher. In sewage samples, there were also abundant *sul* genes with average concentrations ranging from 3.89 × 10^5^ to 4.47 × 10^7^ copies/mL. The concentrations of *sul* genes in animal-derived food samples were at an average level of 1.7 × 104 to 2.75 × 10^6^ copies/g, which was significantly lower than that in sewage and fecal samples. In contrast, the concentration of *sul* genes in surface water was the lowest, ranging from 3.55 × 10^2^ to 1.70 × 10^5^ copies/mL. Furthermore, the average concentrations of *sul1* and *sul2* were much higher than those of *sul3* and *sul4* in all samples, and their average concentrations were 9.78 × 10^6^ copies/g, 5.75 × 10^6^ copies/g, 2.40 × 10^4^ copies/g and 5.49 × 10^4^ copies/g, respectively. In general, the concentrations of *sul* genes are relatively high in samples with abundant microorganisms, such as human feces and sewage. As mentioned above, there are disparities in the positive detection rates and concentrations of *sul* genes among different types of samples. It is necessary to strengthen the monitoring of *sul* genes and explore the potential reasons of these distinctions.

### 3.5 Comparison of the quadruple qPCR and quadruple ddPCR methods for detecting *sul* genes

A comparison was made between the performance of quadruple qPCR and quadruple ddPCR, when serially diluted plasmid solutions of *sul* genes were being detected. (The procedure of the quadruple qPCR method and the standard curve are in the Supplementary Figure 1). Clearly, quadruple ddPCR had higher sensitivity and was capable of detecting single-digit copy samples, while qPCR had a broader quantitative range (Supplementary Table 3). Additionally, the results of 115 diverse samples using two quadruple methods were presented in Table 3. The total positive rate of the ddPCR method (90.43%) was higher than that of the qPCR method (83.40%). Moreover, the McNemar’s chi-square test was used to determine whether the difference in the total positive rate of two methods was statistically significant. The results showed that *p* < 0.05, indicating quadruple ddPCR was more sensitive than quadruple qPCR. Simultaneously, to evaluate the correlation between the two methods, the Cohen’s kappa coefficient was calculated (*K* = 0.681), which suggested a relatively high correlation in the detection results of the two methods.

**Table 3:** The detection results of quadruple qPCR and quadruple ddPCR for *sul* genes in diverse samples.

Genes	Quadruple qPCR			Quadruple dd PCR		
	P	N	Positive rate (%)	P	N	Positive rate (%)
*sul1*	115	0	100	115	0	100
*sul2*	113	2	98.26	114	1	99.13
*sul3*	98	19	85.22	108	7	93.91
*sul4*	76	39	66.87	79	36	68.70
Total	402	58	83.40	416	44	90.43

P denotes the number of positive results, and N is the number of number of negative results.

## 4 Discussion

Several reported methods for quantifying *sul* genes are listed in Table 4. Compared with qPCR, this quadruple ddPCR method has a lower LOD. In contrast to existing ddPCR methods, it can detect more types of *sul* genes. Moreover, the sample types detected in this study are the most extensive. To our knowledge, this is the first application of ddPCR to develop a method for quantifying all *sul* genes, which has proven to exhibit superior sensitivity and comprehensiveness. Furthermore, it had been successfully applied in 115 samples, including human feces, animal-derived food products, sewage, and surface water, thereby demonstrating its robust quantitative performance across diverse matrices.

**Table 4:** The comparison of this study with other methods for quantification of *sul* genes.

Author (year)	Detection method	*sul* genes	Sensitivity	Sample type
Cavé et al. (2016)	ddPCR	*sul1*	1.6 copies	Soil, organic residues
Gong et al. (2018)	LAMP	*sul1*, *sul2*, *sul3*	0.5 pg/reaction	*Enterobacteriaceae*
Di Cesare et al. (2018)	ddPCR	*sul2*	0.21 copies/μL	Marine plankton
Li et al. (2019)	qPCR	*sul1*, *sul2*, *sul3*	10^2^ CFU/mL	*Stenotrophomonas maltophilia*
Xu et al. (2020)	Luminex xTAG	*sul1*, *sul2*, *sul3*, *sul4*	10^1^–10^3^ copies/μL	*Escherichia coli*, *Salmonella*
Kimbell et al. (2021)	ddPCR	*sul1*	3 copies/μL	Microbial communities in cast-iron water pipes
Catania et al. (2024)	Luminex xMAP	*sul1*, *sul2*, *sul3*, *sul4*	10^1^–10^3^ copies/μL	*Escherichia coli*
Sfragano et al. (2024)	Microfluidic card-based electrochemical assay	*sul1*, *sul4*	44.2–48.5 pmol /L	*Escherichia coli*
Gobbo et al. (2024)	ddPCR	*sul2*	13.3 copies	Sewage
This study	ddPCR	*sul1*, *sul2*, *sul3*, *sul4*	3.98–6.16 copies/reaction	Human feces, sewage, urban surface water, and food

During the construction of a multiplex digital PCR system, the design of primers and probes is of great significance. It can directly affect the PCR amplification conditions, the fluorescence intensity of droplets, as well as the distribution of droplet clusters (Su et al., 2024). Firstly, the similarity of the annealing temperatures of primers and probes for each targeted gene is of crucial importance (Svetina et al., 2024). Secondly, the lengths of amplification products for each target gene should not vary greatly and preferably lie in the range of 60–200 bp. Besides, in the development of multiplex ddPCR method, clearly separating all droplet clusters is a significant challenge. To a certain extent, incrementing the concentration of primers and probes specific to the target genes can enhance the corresponding fluorescence amplitude, thus facilitating the differentiation of the target droplet clusters from other clusters. However, if the concentration of primers and probes is too high, the droplet clusters are prone to exhibit “rain” (droplets with intermediate fluorescence intensities do not clearly cluster with either the clearly negative or positive partitions), which is harmful for accurate identification and analysis (Vynck et al., 2023). Therefore, the selection of suitable concentrations of primers and probes is crucial for the successful establishment of a multiplex ddPCR method. As shown in Figure 4, through a series of attempts with different primers and probes combinations, the finally adopted combination parameters are as follows: the primer concentration is set at 700 nM, the ratio of *sul1* to *sul4* is set at 3:10 and the ratio of *sul2* to *sul3* is set at 2:5. With this meticulously chosen combination of conditions, the quadruple ddPCR method separates 16 droplet clusters successfully.

On the whole, compared with other samples, the concentrations of *sul* genes in human feces and sewage were significantly higher. These samples were rich in antibiotic-resistant bacteria and been regarded as reservoirs of ARGs (Leão et al., 2023; Ye et al., 2024). While making a comparison of the concentrations of the four *sul* genes, it was obvious that the earlier discovered *sul1*, *sul2*, and *sul3* are much higher, these results were consistent with a previous study (Hao et al., 2019; Du et al., 2014; Lu et al., 2019). In addition, this study provided data on the positive rates and concentrations of *sul4* in various samples, which had been rarely reported. Among 115 samples, 79 *sul4* positive samples were detected, they mainly derived from sewage, animal-derived food and surface water. By contrast, the positive frequency of *sul4* was relatively low in human feces, with only six positive cases out of 40 samples. It may indicate that the origin of *sul4* in the environment might not be human beings (Rui and Qiu, 2024), or it could just be a bias caused by regional differences (Devanathan et al., 2024; Sharif et al., 2020; Hutinel et al., 2022). As such, it is necessary to enhance the tracking of the novel *sul4*, deeply explore its transmission routes and mechanisms, so as to control the migration of *sul4* from the environment to the human body, reduce the potential risks it may pose to human health.

An efficient method for detecting *sul* genes was developed, which demonstrates great potential and facilitates further research on *sul* genes and the control of their dissemination. Although 115 various samples were detected in this study, it is insufficient to characterize the distribution of *sul* genes. Therefore, it is essential that research on *sul* genes continues in a sustained and long-term manner in the future (Shin et al., 2022).

## 5 Conclusion

For the first time, an innovative quadruple ddPCR method for quantifying all *sul* genes was developed, which was the most comprehensive and sensitive approach for the precise quantification of *sul1*, *sul2*, *sul3*, and *sul4*. It exhibited high sensitivity and good repeatability and allowed for direct quantification of *sul* genes without standard curve. Using this method, the positive rates and concentrations of *sul* genes were obtained from 115 diverse samples, including human feces, animal-derived foods, sewage, and surface water. It turned out that the distribution patterns of four *sul* genes differed among various samples; Specifically for the *sul4*, which was rarely detected in human fecal samples. Overall, the quadruple ddPCR method established in this study can reliably detect and analyze *sul* genes, undoubtedly becoming an efficient means for *sul* genes monitoring. What’s more, this advancement can not only offer technical support for the research on the dissemination of *sul* genes and pollution control, but also provide a reference for the establishment of detection methods for other ARGs. This study complied with the dMIQE guidelines (Minimum Information for Publication of Quantitative Digital PCR Experiments for 2020).

## Data Availability

The original contributions presented in the study are included in the article/Supplementary material, further inquiries can be directed to the corresponding authors.
