# Supplementary material for: Development and application of a highly sensitive quadruple droplet digital PCR method for simultaneous quantification of sulfonamide resistance genes
Source: Front Microbiol. 2025 May 21;16:1612740. doi: 10.3389/fmicb.2025.1612740 (PMC12133762; doi:10.3389/fmicb.2025.1612740)
Supplement: Supplementary file 1 [file Table_1.docx]

Supplementary Material

**Development and application of a highly sensitive quadruple droplet digital PCR method for simultaneous quantification of sulfonamides resistance genes**

# The experimental procedure of PCR

The reaction system of PCR consists of 25 μL of 2×TaKaRa Taq HS Perfect Mix (Takara Bio Inc, Japan), 5 μL of primers (10μM) and 5 μL of template DNA, and 10 μL nuclease-free water is supplemented to make a total volume of 50 μL. Amplification conditions, heating for 3 min at 94 ℃, followed by 30 cycles at 94 ℃ for 5 s, hold for 5 s under the appropriate annealing temperature, and 68 ℃ for 20 s. The primers and experimental conditions of the first-generation PCR are presented as follows (Supplementary Table 1).

**Supplementary Table 1**. The primers of PCR

| Gene | Primer | Sequence 5’- 3’ | Product size(bp) | annealing temperatue (℃） | References |
| --- | --- | --- | --- | --- | --- |
| *sul1* | *sul1*-F | GGCCGATGAGATCAGACGTA | 413 | 57 | Jiang et al. (2019) |
|  | *sul1*-R | TTTGAAGGTTCGACAGCACG |  |  |  |
| *sul2* | *sul2*-F | GCAGGCGCGTAAGCTGA | 675 | 60 | Jiang et al. (2019) |
|  | *sul2*-R | GGCTCGTGTGTGCGGATG |  |  |  |
| *sul3* | *sul3*-F | GAGCAAGATTTTTGGAATCG | 750 | 56 | Hammerum et al. (2006) |
|  | *sul3*-R | CTAACCTAGGGCTTTGGATAT |  |  |  |
| *sul4* | *sul4*-F | ATGTCAACCACACTAACCAGCTT | 858 | 55 | Shindoh et al. (2023) |
|  | *sul4*-R | AGCACTGAAATCCTTTAACGTCTC |  |  |  |

# Development of quadruple qPCR method.

First of all, according to the melting temperature of the primers and probes (Supplementary Table 2), a series of temperature gradients were set up to determine the optimal annealing temperature of each target. Then, the primers and probes of the four target genes were combined pairwise for qPCR amplification to confirm no mutual interference. Later, different combinations of primers and probes were designed, the optimal multiplex qPCR system was selected based on a lower Ct value and a higher fluorescence intensity in the amplification curve. The methodology is presented as follows: the multiplex qPCR mixture contained 10μL 2× Premix Ex Taq(Takara Bio Inc, Japan), 0.3 μL each primer (20 μM), 0.3 μL sul1 probe (10 μM,FAM-labled), 0.4 μL sul2 probe (10 μM, HEX-labled), 0.5 μL sul3 probe (10 μM, CY5-labled), 0.3 μL sul4 probe (10 μM, ROX-labled), 2 μL DNA sample, and 4 μL nuclease-free water to a final volume of 20 μL. All reactions were amplified on a Applied BiosystemsTM 7500 Real-time System (Thermo Scientific, USA) at 95℃ for 30 s, followed by 40 cycles of 95℃ for 5 s and 57℃ for 30 s.

The standard curves of *sul* genes were generated using the gradient-diluted plasmid mentioned above, the best linear fitting effect is in the range of 107 to 101 copies/μL. Ideal qPCR methods demand that the correlation coefficient(R2) > 0.990, the slope of the standard curve ranges from -3.1 to -3.6, and the amplification efficiency range (Eff%) between 90%~ 110%. The multiplex qPCR system in this study fully complies with the requirements (supplementary Figure 1).

**Supplementary Table 2**. The primers and probes for quadruple qPCR.

| Gene | Gene bank number and position | Primer/  Probe | Sequence 5’- 3’ | Product size (bp) | References |
| --- | --- | --- | --- | --- | --- |
| *sul1* | JF969163.1;  1054-1893 | *sul1*-F | CCGTTGGCCTTCCTGTAAAG | 67 | Han et al. 2021 |
|  |  | *sul1*-R | TTGCCGATCGCGTGAAGT |  |  |
|  |  | *sul1*-P | FAM-CAGCGAGCCTTGCGGCGG-BHQ1 |  |  |
| *sul2* | AY055428.1;  20269-21084 | *sul2*-F | CGGCTGCGCTTCGATT | 60 | Han et al. 2021 |
|  |  | *sul2*-R | CGCGCGCAGAAAGGATT |  |  |
|  |  | *sul2*-P | HEX-CGGTGCTTCTGTCTGTTTCGCGC-BHQ1 |  |  |
| *sul3* | NZ_NIYS01000141.1;  2098-2889 | *sul3*-F | AGGCTTGGCAAAGTCAGATTG | 68 | This study |
|  |  | *sul3*-R | TAGTAGCTGCACCAATTCGC |  |  |
|  |  | *sul3*-P | CY5-ACTTGTGTTGATGCACTCCGTT-BHQ2 |  |  |
| *sul4* | NG_056174.1  1-1064 | *sul4*-F | CGCGCAAATCATTTATTGGCTA | 68 | This study |
|  |  | *sul4*-R | GGCCGCCGTTCCTTCTA |  |  |
|  |  | *sul4*-P | ROX-ACGCTCGATTTGCCGCCAGACCAG-BHQ2 |  |  |

F, forward primer; R, reverse primer; P, probe; FAM, fluorescein amidite; HEX, hexachloro fluorescein;

CY5, cyanine 5; ROX, carboxy-X-rhodamine; BHQ1, black hole quencher 1; BHQ2, black hole quencher 2. FAM, HEX, CY5, and ROX are reporter groups that modify probes, while BHQ1 and BHQ2 are quencher groups that modify probes.

| 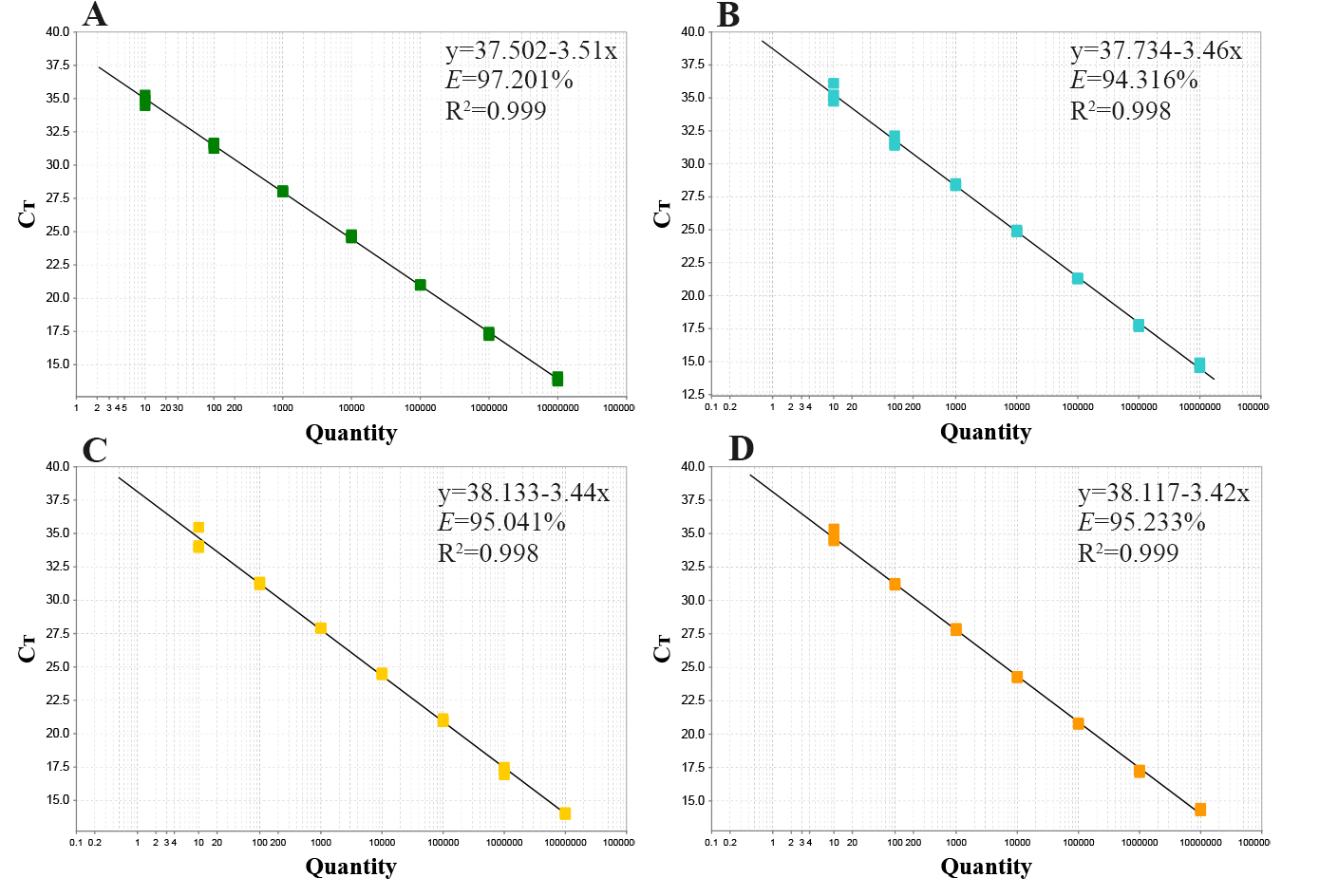  **Supplementary Figure 1.** Standard curve of quadruple qPCR: (A) *sul1* gene; (B) *sul2* gene; (C) *sul3* gene; (D) *sul4* gene. |
| --- |

### The results of Sanger sequencing

| 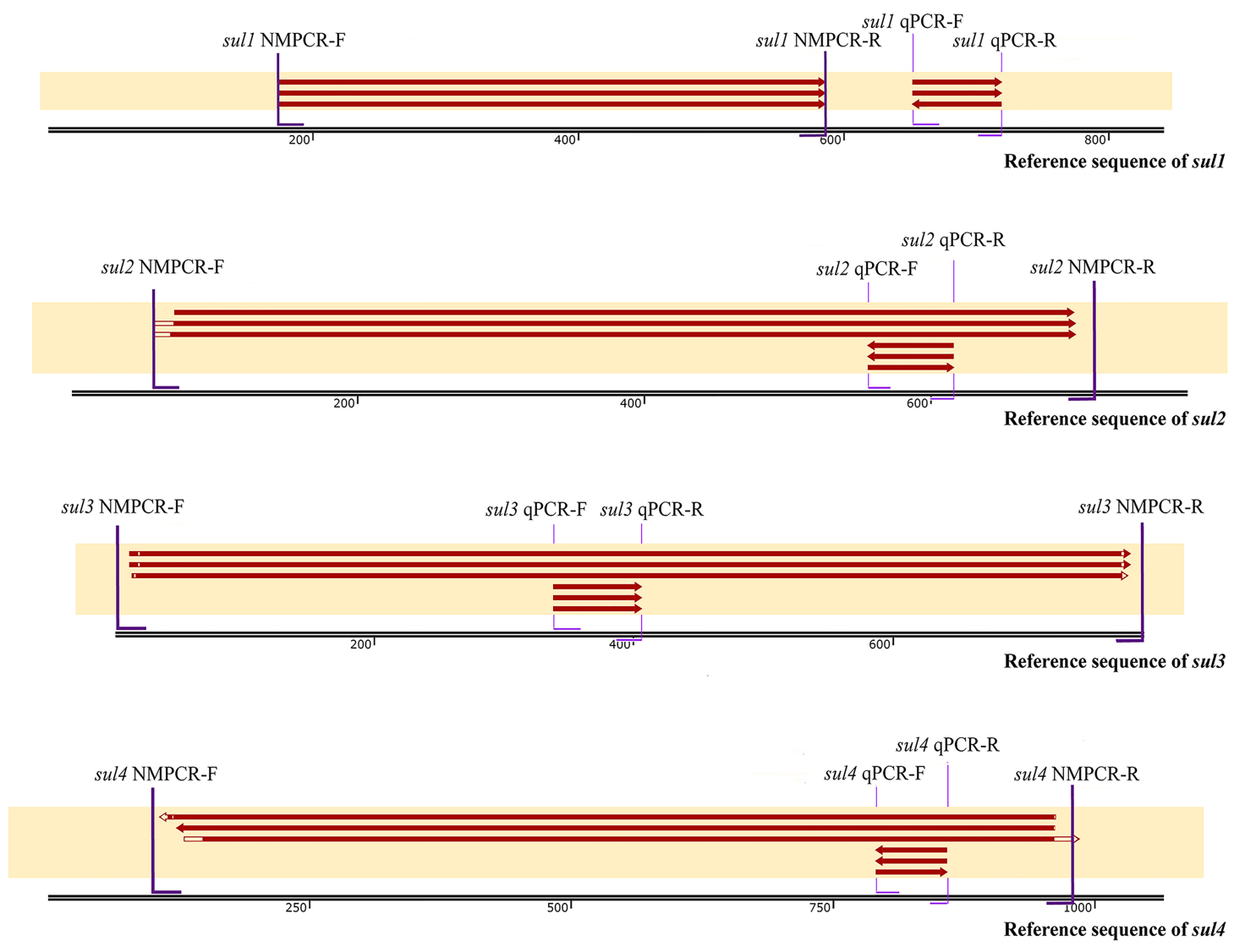  **Supplementary Figure 2.** Analyzing the Sanger sequencing results of PCR and qPCR: the black parallel lines signify the reference sequence of the target gene. NMPCR is PCR, and F and R refer to the forward and reverse primers. The PCR and qPCR products are located in the middle of the corresponding primer sequences. The red solid line represents the portion of the PCR product sequence that is completely identical to the reference sequence. |
| --- |

# Comparison of quadruple qPCR and quadruple ddPCR

**Supplementary Table 3**. Detection of serially diluted plasmid solutions using quadruple qPCR and quadruple ddPCR**.**

| Target genes | Plasmid solutions  concentration  (copies/μL) | 4-plex-qPCR | | 4-plex ddPCR | |
| --- | --- | --- | --- | --- | --- |
|  |  | Ct ($\bar{x}$ ± s) | CV(%) | copies/reaction ($\bar{x}$ ± s) | CV(%) |
| *sul1* | 10^7^ | 13.91 ± 0.17 | 1.22 | H |  |
|  | 10^6^ | 17.32 ± 0.13 | 0.75 | H |  |
|  | 10^5^ | 20.99 ± 0.02 | 0.10 | H |  |
|  | 10^4^ | 24.62 ± 0.16 | 0.65 | (6.21 ± 0.21)×10^4^ | 3.38 |
|  | 10^3^ | 28.03 ± 0.05 | 0.18 | (5.51 ± 0.38)×10^3^ | 6.90 |
|  | 10^2^ | 31.49 ± 0.21 | 0.67 | (5.52 ± 0.48)×10^2^ | 8.70 |
|  | 10^1^ | 34.88 ± 0.39 | 1.12 | (5.84 ± 0.30)×10^1^ | 5.14 |
|  | 10^0^ | L |  | (5.65 ± 1.20) | 24.77 |
| *sul2* | 10^7^ | 13.88 ± 0.19 | 1.37 | H |  |
|  | 10^6^ | 16.90 ± 0.09 | 0.53 | H |  |
|  | 10^5^ | 20.42 ± 0.17 | 0.83 | H |  |
|  | 10^4^ | 24.01 ± 0.04 | 0.17 | (7.00 ± 0.26)×10^4^ | 3.71 |
|  | 10^3^ | 27.51 ± 0.08 | 0.29 | (6.38 ± 0.34)×10^3^ | 5.30 |
|  | 10^2^ | 30.73 ± 0.27 | 0.88 | (6.73 ± 0.45)×10^2^ | 6.69 |
|  | 10^1^ | 34.42 ± 0.69 | 2.00 | (6.41 ± 0.55)×10^1^ | 8.58 |
|  | 10^0^ | L |  | (7.11 ± 1.36) | 19.13 |
| *sul3* | 10^7^ | 14.02 ± 0.07 | 0.50 | H |  |
|  | 10^6^ | 17.23 ± 0.28 | 1.62 | H |  |
|  | 10^5^ | 21.00 ± 0.12 | 0.57 | H |  |
|  | 10^4^ | 24.49 ± 0.07 | 0.29 | (6.01 ± 0.17)×10^4^ | 2.83 |
|  | 10^3^ | 27.91 ± 0.02 | 0.72 | (5.21 ± 0.36)×10^3^ | 6.91 |
|  | 10^2^ | 31.29 ± 0.11 | 0.35 | (5.31 ± 0.26)×10^2^ | 4.90 |
|  | 10^1^ | 34.50 ± 0.82 | 2.38 | (4.40 ± 0.38)×10^1^ | 8.64 |
|  | 10^0^ | L |  | (5.39 ± 1.38) | 24.60 |
| *sul4* | 10^7^ | 14.34 ± 0.10 | 0.70 | H |  |
|  | 10^6^ | 17.24 ± 0.10 | 0.10 | H |  |
|  | 10^5^ | 20.78 ± 0.05 | 0.24 | H |  |
|  | 10^4^ | 24.26 ± 0.05 | 0.21 | (7.31 ± 0.27)×10^4^ | 3.69 |
|  | 10^3^ | 27.83 ± 0.08 | 0.29 | (6.60 ± 0.26)×10^3^ | 3.94 |
|  | 10^2^ | 31.21 ± 0.07 | 0.22 | (6.49 ± 0.24)×10^2^ | 3.70 |
|  | 10^1^ | 34.80 ± 0.47 | 1.36 | (5.67 ± 0.48)×10^1^ | 8.47 |
|  | 10^0^ | L |  | (6.16±0.94) | 15.26 |

L represents concentrations lower than the detection limit, H indicates that the detected concentration is higher than the upper detection limit of the instrument.

| 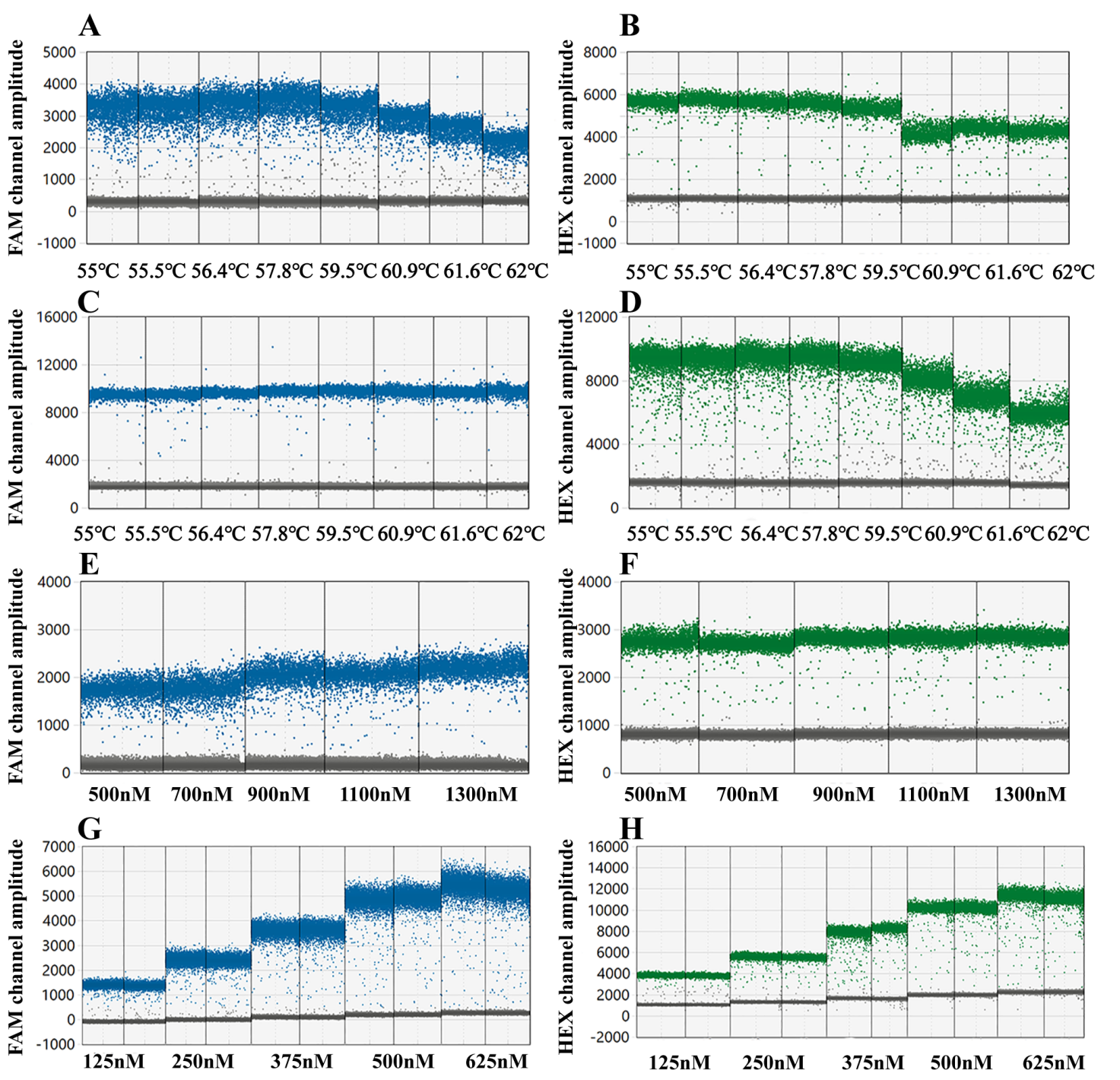  **Supplementary Figure 3 .** Method optimizing for one target detection: (A~D) show the amplitude of the *sul1*, *sul2*, *sul3* and *sul4* varying with temperature. (E) the influence of the primer concentration of *sul1* on the fluorescence amplitude; (F) the influence of the primer concentration of *sul2* on the fluorescence amplitude; (G) the effect of the probe concentration of *sul1* on the fluorescence amplitude; (H) the effect of the probe concentration of *sul2* on the fluorescence amplitude. Blue indicates FAM-labeled positive droplets, green indicates HEX-labeled positive droplets, and gray indicates negative droplets. |
| --- |

## References

Hammerum, Anette M., Sandvang, Dorthe, Andersen, Sigrid R., Seyfarth, Anne Mette, Porsbo, Lone Jannok, Frimodt-Møller, Niels, et al. (2006). Detection of sul1, sul2 and sul3 in sulphonamide resistant Escherichia coli isolates obtained from healthy humans, pork and pigs in Denmark. *Int. J. Food Microbiol.* 106, 235-237. doi: 10.1016/j.ijfoodmicro.2005.06.023.

Han, H., Bai, M., Chen, Y., Gong, Y., Wu, M., Yang, H., et al. (2021). Dynamics of Diversity and Abundance of Sulfonamide Resistant Bacteria in a Silt Loam Soil Fertilized by Compost. *Antibiotics (Basel).* 10: 699. doi: 10.3390/antibiotics10060699.

Jiang, H., Cheng, H., Liang, Y., Yu, S., Yu, T., Fang, J., et al. (2019). Diverse Mobile Genetic Elements and Conjugal Transferability of Sulfonamide Resistance Genes (sul1, sul2, and sul3) in Escherichia coli Isolates From Penaeus vannamei and Pork From Large Markets in Zhejiang, China. *Front. Microbiol.* 10:1787. doi: 10.3389/fmicb.2019.01787.

Shindoh, S., Kadoya, A., Kanechi, R., Watanabe, K., and Suzuki, S. (2023). Marine bacteria harbor the sulfonamide resistance gene sul4 without mobile genetic elements. *Front. Microbiol.* 14: 1230548. doi: 10.3389/fmicb.2023.1230548.
